# Temporoparietal Connectivity Within Default Mode Network Associates With Clinical Improvements in Schizophrenia Following Modified Electroconvulsive Therapy

**DOI:** 10.3389/fpsyt.2021.768279

**Published:** 2022-01-04

**Authors:** Qiang Hu, Huan Huang, Yuchao Jiang, Xiong Jiao, Jie Zhou, Yingying Tang, Tianhong Zhang, Junfeng Sun, Dezhong Yao, Cheng Luo, Chunbo Li, Jijun Wang

**Affiliations:** ^1^Shanghai Key Laboratory of Psychotic Disorders, Shanghai Mental Health Center, Shanghai Jiao Tong University School of Medicine, Shanghai, China; ^2^The Clinical Hospital of Chengdu Brain Science Institute, MOE Key Lab for Neuroinformation, High-Field Magnetic Resonance Brain Imaging Key Laboratory of Sichuan Province, School of Life Science and Technology, University of Electronic Science and Technology of China, Chengdu, China; ^3^School of BIomedical Engineering, Shanghai Jiao Tong University, Shanghai, China; ^4^CAS Center for Excellence in Brain Science and Intelligence Technology (CEBSIT), Chinese Academy of Science, Shanghai, China; ^5^Brain Science and Technology Research Center, Shanghai Jiao Tong University, Shanghai, China; ^6^Institute of Psychology and Behavioral Science, Shanghai Jiao Tong University, Shanghai, China

**Keywords:** schizophrenia, modified electroconvulsive therapy, default mode network, functional connectivity, longitudinal study

## Abstract

Although modified electroconvulsive therapy (ECT) has been reported to be effective for the treatment of schizophrenia (SCZ), its action mechanism is unclear. To elucidate the underlying ECT mechanisms of SCZ, this study used a longitudinal cohort including 21 SCZ patients receiving only antipsychotics (DSZ group) and 21 SCZ patients receiving a regular course of ECT combining with antipsychotics (MSZ group) for 4 weeks. All patients underwent magnetic resonance imaging (MRI) scans at baseline (t1) and follow-up (t2) time points. A matched healthy control (HC) group included 23 individuals who were only scanned at baseline. Functional connectivity (FC) within the default mode network (DMN) was evaluated before and after ECT. Significant interaction of the group over time was found in FC between angular gyrus (AG) and middle temporal gyrus (MTG). *Post-hoc* analysis showed a significantly enhanced FC of left AG(AG.L) and right MTG (MTG.R) in the MSZ group relative to the DSZ group. In addition, the right AG (AG.R) showed significantly enhanced FC between MTG.R and left MTG (MTG.L) after ECT in the MSZ group, but no in the DSZ group. In particular, the FCs change in AG.L-MTG.R and AG.R-MTG.R were positively correlated with the Positive and Negative Syndrome Scale (PANSS) negative score reduction. Furthermore, the FC change in AG.L-MTG.R was also positively correlated with the PANSS general psychopathology score reduction. These findings confirmed a potential relationship between ECT inducing hyperconnectivity within DMN and improvements in symptomatology of SCZ, suggesting that ECT controls mental symptoms by regulating the temporoparietal connectivity within DMN.

## Introduction

Schizophrenia (SCZ) is a severe mental illness that is debilitating for affected individuals and imposes a great burden on caregivers (1). Due to the heterogeneity of the manifestations of SCZ, we can divide SCZ syndrome into three subtypes, including positive symptoms (delusions, hallucinations, positive formal thought disorder, and persistently bizarre behavior), negative symptoms (affective flattening, alogia, avolition, anhedonia, and attentional impairment) and mixed SCZ (not meet criteria for either positive or negative SCZ, or meet criteria for both) (2). While psychopharmacotherapy has been the main treatment for SCZ, modified electroconvulsive therapy (ECT) presents an effective alternate treatment for patients with various SCZ-like conditions including psychotic disorders and catatonia (3, 4), and has been reserved for medication-resistant and severe cases of SCZ (5). Modified ECT, combined with anesthetics, is now a common treatment for patients with intractable mental illness that cannot be controlled by other methods. Compared with unmodified ECT, the use of muscle relaxants and anesthetics effectively safeguards the patient, prevented fractures, provided complete loss of consciousness and minimal anticonvulsant adverse reactions, prevented hypoxia, and managed ECT adverse reactions (6). Evidence suggests that combined ECT and antipsychotics was superior to medication alone (7).

Resting-state functional magnetic resonance imaging (fMRI) has become available in the past decades for the study *in vivo* of psychopathology and treatment response in severe mental illnesses, based on its sensitivity for the detection of psychosis and treatment responses (8). Among numerous fMRI studies on ECT for psychiatric disorders, the main topic has been major depressive disorder (MDD) (8), with a few studies hitherto exploring the neurophysiological mechanism and relationship of the resting state to symptomatology of SCZ. Functional connectivity (FC) measures, defined as instantaneous, zero-time lagged temporal correlations between spatially distinct neurophysiologic events (9), have been investigated in studies of SCZ by different data-driven algorithms (10–16). One study reported on the effects of right unilateral ECT on brain structure and function among SCZ and MDD patients (8), which found that ECT had similar effects on brain volume in SZ and MDD patients, with increased gray matter volume in the right amygdala, hippocampus and insula. In contrast to the volume study results, functional data showed a decrease in FC between the right amygdala and other brain regions after ECT in MDD, including the temporoparietal, prefrontal and cortical midline structures, whereas FC between the right amygdala and hypothalamus increased in SZ. A number of research teams have identified functional dysconnectivity within and across many networks and/or pathways, such as the thalamocortical circuits (11) and the default mode network (DMN) (12) using seed-based connectivity algorithm. Of note, the study of Bluhm et al. first reported abnormal temporal coherence of BOLD signals in DMN-associated regions in SCZ with a seed-based connectivity algorithm (17). Specifically, FCs between the posterior cingulate cortex (PCC) and the medial prefrontal lobe, lateral parietal lobe, and cerebellum were reduced in SCZ in this study. However, many subsequent studies consistently found increased FC within the DMN of SCZ, both in high-risk in psychosis patients (18) and in first-episode patients (19). Furthermore, in a review, Fornito and colleagues suggested that SCZ at early stages presented stronger FC, with a generally declining FC decrease characterizing later illness stages. The shift in FC across different stages of SCZ implies an association between decreasing FC and illness deterioration (20). We reported in a previous study of medication-resistant, chronically ill SCZ patients that ECT “normalized” global FC intensity within the previously disrupted DMN, indicating a possibly important neural mechanism of ECT in the treatment of SCZ (21).

Though previous evidence (22, 23) has shown that DMN intensity tends to normalize after ECT in SCZ, to our best knowledge, little is known about the effect of ECT on DMN connectivity in relation to the clinical symptoms. Therefore, we conducted a study in effort to link the change in FC at ECT to clinical phenomenology. We hypothesize that the FC changes in SCZ patients after receiving ECT would be associated with improvement in psychopathology scores of SCZ.

## Materials and Methods

### Participants

**Figure 1:**
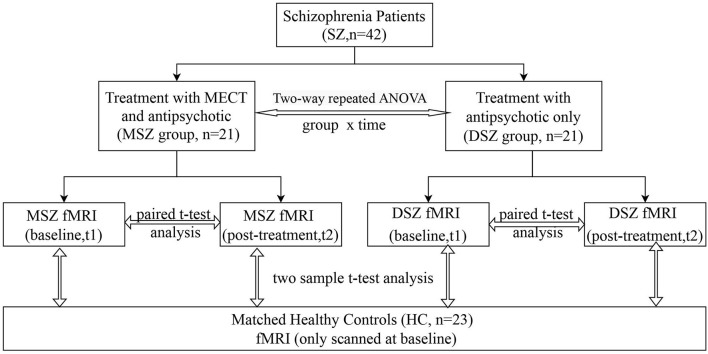
Flow chart. 42 patients were divided into two groups according to their treatment strategy. One group (MSZ, *n* = 21) received 4-weeks of ECT together with anti- psychotics; the other (DSZ, *n* = 21) was treated only with antipsychotics. 23 healthy controls were also included. Structural and functional MRI were scanned twice at baseline and post-treatment.

In this study (Figure 1), we recruited forty-two patients with SCZ who were assigned to two subgroups according to their treatment strategy. All patients were recruited from inpatients at the Shanghai Mental Health Center (SMHC). Among them, 21 cases with previous treatment history were determined to be drug-resistant because they had not responded to two or more adequate antipsychotic trials in the past five years (24), and the patients and their families provided informed consent for ECT. After drug-resistance was diagnosed and the ECT course was voluntarily selected, the patients were given ECT in combination with antipsychotic medications for 4 weeks (MSZ group). In addition, all patients in the MSZ group met ECT criteria and had no ECT treatment history in the past 6 months. Of the 21 patients who were drug-free or did not agree to receive ECT, these patients only received antipsychotic treatment, i.e., treatment as usual (DSZ group). The diagnosis of SCZ was according to the SCID-I/P (Structural Clinical Interviewfor DSM-IV-TR, Patient's version) criteria. Clinical symptomatic severity was evaluated by the Positive And Negative Syndrome Scale (PANSS) (25) and all patients had a total PANSS score of more than 60. All patients were treated with antipsychotics and daily antipsychotic doses were converted to chlorpromazine equivalent (mg/d) (26) shown in Table 1. In both MSZ and DSZ groups, each patient's antipsychotic regimen remained the same during the study period as before. Most of these patients were prescribed atypical antipsychotics (Table 2). Specifically, in the MSZ group, 3 were taking one antipsychotic, 12 were taking two antipsychotics, and 6 were taking three antipsychotics. Of 21 patients in the DSZ group, 10 were taking one antipsychotic, 10 were taking two antipsychotics and 1 was taking three antipsychotics. Additionally, we recruited 23 healthy subjects as a healthy control group (HC group), who were matched to both patient groups with respect to age, gender, and education (Table 1). All participants ranged in age from 17 to 46 and were right-handed. The exclusion criteria for all participants included history of major medical or neurological illness, organicmental disorders, traumatic brain injury, dementia, substance abuse or dependence, and MRI contraindications. Details of participant demographics are available in our previous study with the same cohort (27). The Ethics Committee of SMHC approved the study protocol; all subjects provided written, informed consent prior to participation in the study.

**Table 1:** Demographic and clinical data of participants.

**Characteristic**	**MSZ (*n =* 21)**	**DSZ (*n =* 21)**	***P*-value^a^**	**HC (*n =* 23)**
	**mean (SD)**	**mean (SD)**		**mean (SD)**
Gender (M/F)^b^	10/11	9/12	0.757	11/12
Age (years)	29.2(7.1)	30.7(7.8)	0.524	31.2 ± 5.9
Education (years)	12.3(3.4)	12.6(2.9)	0.773	13.5 ± 2.5
Handness (left/right)^b^	0/21	0/21	1.000	0/23
Chinese Han nationality^b^	21	21	1.000	23
Married/unmarried/divorced^b^	5/15/1	5/14/2	0.285	13/10/0
Smoking/non-smoking^b^	3/18	3/18	1.000	7/16
Drinking/non-drinking^b^	0/21	0/21	1.000	3/20
Family history of schizophrenia (yes/no)^b^	8/13	6/15	0.513	0/23
Illness duration (months)^c^	79.8(54.4)	78.7(80.9)	0.435	–
Interval of scans (days)	36.1(10.2)	35.3(14.6)	0.827	–
Chlopromazine equivalents (mg/d)^c^	604.6(565.6)	532.6(461.2)	0.504	–
**Baseline PANSS score**				
Total	71.6(8.4)	70.8(9.7)	0.673	–
Positive	20.7(2.6)	19.1(3.5)	0.107	–
Negative	19.3(7.4)	17.4(5.1)	0.339	–
General	32.0(3.8)	34.2(5.7)	0.139	–
**4-weeks PANSS score**				
Total	49.7(9.6)	50.5(12.6)	0.816	–
Positive	10.9(3.0)	12.0(4.7)	0.375	–
Negative	14.6(6.1)	14.0(5.3)	0.768	–
General	24.3(3.33)	24.5(5.4)	0.891	–

a *P-values were obtained using two sample t-tests except where noted.*

b *P-values were obtained using the chi-square test.*

c *P-values were obtained using the Mann-Whitney tests as a result of the substantial variability in each group*.

**Table 2:** Information on antipsychotic medication usage for each patient.

**Subject**	**Group**	**Drug 1**	**Average dose (mg)**	**Drug 2**	**Average dose (mg)**	**Drug 3**	**Average dose (mg)**	**CPZ equivalent units**	**Number of drug types**
Sub1	ECT	Risperidone	3	–	–	–	–	150	1
Sub2	ECT	Olanzapine	5	Paliperidone ER	6	–	–	250	1
Sub3	ECT	Amisulpride	900	Aripiprazole	10	Clozapine	200	2,256.8	3
Sub4	ECT	Clozapine	75	Aripiprazole	12.5	–	–	316.7	1
Sub5	ECT	Paliperidone ER	6	Haloperidol	9	Quetiapine	150	800	3
Sub6	ECT	Risperidone	3	Quetiapine	100	–	–	283.3	1
Sub7	ECT	Risperidone	5	Olanzapine	20	–	–	650	1
Sub8	ECT	Risperidone	1	Paliperidone ER	4.5	–	–	162.5	1
Sub9	ECT	Paliperidone ER	3	Clozapine	25	Amisulpride	400	891	3
Sub10	ECT	Olanzapine	20	Haloperidol	5	–	–	650	1
Sub11	ECT	Risperidone	3	Olanzapine	7.5	–	–	300	1
Sub12	ECT	Olanzapine	20	Perphenazine	8	–	–	480	1
Sub13	ECT	Chlorpromazine	250	Paliperidone ER	3	–	–	325	1
Sub14	ECT	Ziprasidone	80	Olanzapine	7.5	–	–	283.4	1
Sub15	ECT	Risperidone	4	–	–	–	–	200	1
Sub16	ECT	Ziprasidone	80	Quetiapine	300	Amisulpride	600	1,682.3	3
Sub17	ECT	Risperidone	2.5	Olanzapine	10	–	–	325	1
Sub18	ECT	Olanzapine	7.5	Ziprasidone	40	Clozapine	200	616.7	3
Sub19	ECT	Olanzapine	15	Amisulpride	600	Risperidone	2	1,549	3
Sub20	ECT	Olanzapine	15	Paliperidone ER	1.5	–	–	337.5	1
Sub21	ECT	Paliperidone ER	7.5	–	–	–	–	187.5	1
Sub22	DRUG	Olanzapine	17.5	Amisulpride	200	Paliperidone ER	4.5	845.5	3
Sub23	DRUG	Risperidone	3	–	–	–	–	150	1
Sub24	DRUG	Olanzapine	5	Risperidone	3	–	–	250	2
Sub25	DRUG	Risperidone	4	–	–	–	–	200	1
Sub26	DRUG	Risperidone	5.5	–	–	–	–	275	1
Sub27	DRUG	Risperidone	4	–	–	–	–	200	1
Sub28	DRUG	Amisulpride	700	–	–	–	–	1,340.5	1
Sub29	DRUG	Ziprasidone	100	Olanzapine	10	–	–	366.7	2
Sub30	DRUG	Clozapine	200	Risperidone	3	–	–	550	2
Sub31	DRUG	Clozapine	100	Risperidone	4	–	–	400	2
Sub32	DRUG	Olanzapine	5	Paliperidone ER	9	–	–	325	2
Sub33	DRUG	Risperidone	4	–	–	–	–	200	1
Sub34	DRUG	Penfluridol	7.14	Quetiapine	350	–	–	1,266.7	2
Sub35	DRUG	Paliperidone ER	3	Aripiprazole	2.5	-	–	108.3	2
Sub36	DRUG	Risperidone	3	–	–	–	–	150	1
Sub37	DRUG	Olanzapine	20	–	–	–	–	400	1
Sub38	DRUG	Aripiprazole	15	–	–	–	–	200	1
Sub39	DRUG	Ziprasidone	120	–	–	–	–	200	1
Sub40	DRUG	Amisulpride	500	Aripiprazole	5	–	–	1,024.2	2
Sub41	DRUG	Amisulpride	800	–	–	–	–	1,532	1
Sub42	DRUG	Quetiapine	900	Magnesium Valproate	625	–	–	1,200	2

### ECT Protocol

Patients received ECT treatment three times per week (Monday, Wednesday, and Friday) for 4 weeks by bilateral electrical stimulation with a Thymatron System IV (Somatics, Lake Bluff, IL, USA). Anesthesia was induced by intravenous etomidate (0.21-0.3 mg/kg) in combination with propofol (1.82–2.44 mg/kg) prior to ECT. In addition, intravenous succinylcholine (1 mg/kg) was administered to reduce the risk of bone fracture or other injury. In addition, we applied intravenous atropine (0.5 mg) to inhibit airway secretion. We chose bitemporal ECT stimulus, which is widely used in clinical practice for MDD and has the advantage of bring rapid clinical remission (28, 29). ECT parameters were as follows: maximum charge delivered, 504 mCoulombs; output current, 0.9 A; frequency, 10–70 Hz; pulse width, 1.0 ms; maximum stimulus duration, 8s. Patients received at least eight rounds of ECT (1 MSZs, 8 times; 3 MSZs, 10 times; 1 MSZ, 11 times; 16 MSZs, 12 times), and the treatment was taken every other day. During the treatment process, the epilepsy waveform was recorded to determine the seizure time and monitoring its evolution, and to make corresponding adjustments (30).

### Data Acquisition

Imaging data were acquired using a 3-T Siemens Magnetom veriosyngo MR B17 scanner. fMRI data were obtained using a gradient echo planar imaging (EPI) sequence (T*R* = 2,000 ms; TE = 30 ms; flip angle = 90°; FOV = 220 mm × 220 mm; matrix = 64 × 64; slice thickness = 4 mm; 30 slices; voxel size = 3.4 × 3.4 × 3.4 mm; 180 volumes). The patients underwent scanning twice at baseline and after the 4-week treatment, while the control subjects were scanned only at baseline. Each patient's initial fMRI scan was obtained within 24 h prior to the first ECT session, and the final fMRI scan was collected 24–48 h after the last ECT session. The participants were instructed to remain awake with eyes closed and to not focus their thoughts on anything in particular.

### FMRI Data Preprocessing

The data preprocessing pipeline was similar to that in a previous study (21). In brief, we removed the first 10 time points to allow for signal equilibrium and to permit the subjects' adaptation to the scanning noise. Next, we performed a slice-timing correction, realignment correction, normalization and resampling to a final voxel size of 3 × 3 × 3 mm^3^. Next, we regressed out the nuisance covariates including 24 head motion parameters (31, 32), white matter (WM) and cerebrospinal fluid (CSF) signals and linear trending, followed by temporal scrubbing (33) and temporal filtering (0.01–0.1 Hz). Finally, the data was smoothed with a Gaussian kernel (FWHM = 6 mm). The differences of head-motion (frame-wise displacement, FD) were assessed using repeated measures ANOVA and *post-hoc* analyses. Data preprocessing was performed in SPM12 (http://www.fil.ion.ucl.ac.uk/spm/software/spm12) and DPABI (http://rfmri.org/dpabisoftware).

### DMN Identification

A seed-based FC analysis was performed to examine the default mode network (DMN) in all subjects. A spherical region (radius 10 mm) located in the posterior cingulate cortex (PCC) (−5, −49, 40) was selected as the seed (34) and the mean BOLD time series were extracted from the seed. Then, we calculated Pearson's correlation coefficients between the averaged time series of the seed and that of all other voxels in the whole brain. Next, we normalized these correlation coefficients to *z*-scores by Fisher's *r*-to-*z* transformation. Thus, a whole brain *z*-score map was created for each subject. One-sample *t*-test was performed across all pre-treatment patients to determine regions that had significant FC with the seed, with the significance level set at *P* < 0.05 with the FWE correction. Six regions of interest (ROIs) for medial prefrontal cortex (MPFC), PCC, bilateral AG and MTG were selected as the DMN nodes, consistent with previous DMN studies (35–37). The 8-mm sphere of each DMN node was extracted for the FC analyses.

### FC Within DMN

To quantify the FC within the DMN, the time series were extracted by averaging the time series of voxels within each ROI of the DMN. The Pearson's correlation coefficients were computed between the any two-time series of ROIs, and Fisher's r-to-z transformation was used to normalize the correlation coefficient.

### Statistical Analysis

Linear mixed model analyses were used to evaluate the effect of group (MSZ vs. DSZ), time (*t*1 vs. *t*2) and group x time interaction on the FC between each pair of ROIs while controlling for age, sex and chlorpromazine equivalent. Time and group were fitted as fixed factors and participant as a random factor. To compare the longitudinal changes between *t*1 and *t*2, paired *t*-test was performed in each patient group. In addition, the differences between each patient group and healthy control group were investigated using two-sample *t*-test, after regressing out the age and gender. To correct for multiple comparisons, we applied a permutation-based procedure for controlling the family wise error rate (35).

### Relationships Between Connectivity Alterations and Clinical Features

The relationships between connectivity alterations and clinical features were analyzed using SPSS 28.0. The FC patterns in the SCZ patients that showed significant longitudinal changes or differences from the healthy controls group were extracted. A FC change was defined as the subtraction of amplitudes between *t*2 and *t*1. As the subtraction results did not conform with normal distribution, we used Spearman correlation analysis to investigate the relationships between FC changes and symptom remissions (PANSS positive, negative, general psychopathology and total scores reduction) in the MSZ group, after regressing out the age, gender and ECT number.

## Results

### DMN Spatial Pattern

**Figure 2:**
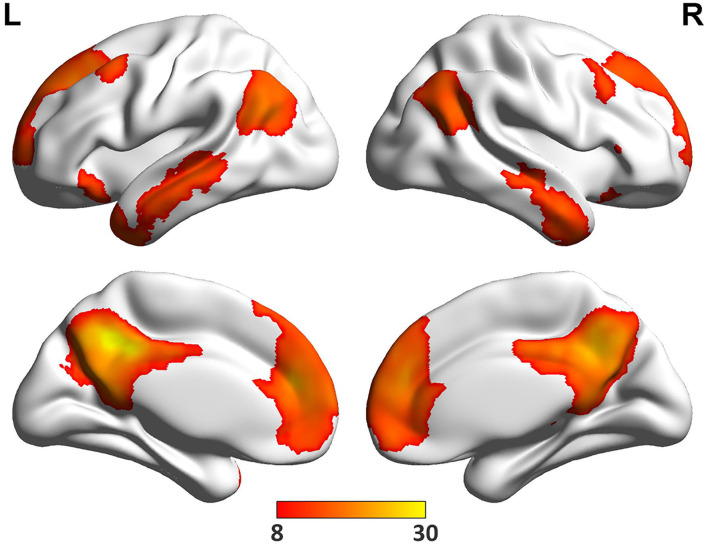
The DMN regions. One sample *t*-test was performed across all pre-treatment patients to determine regions that had significant connectivity with the PCC seed (−5, −49, 40) (*P* < 0.05, FWE correction).

The spatial pattern of DMN was shown in Figure 2. Consistent with previous studies (9–11), six ROIs of MPFC, PCC, bilateral AG and MTG were selected as the DMN nodes (Table 3).

**Table 3:** The DMN regions.

**Region**	**Hemisphere**	**MNI coordinates (*x*, *y*, *z*)**			***T*-value**	**Cluster size (voxels)**
MPFC	L/R	3	45	21	19.63	2,905
PCC	L/R	−6	−48	39	40.72	2,246
AG	L	−45	−60	24	14.52	615
	R	51	−54	27	12.29	423
MTG	L	−48	−15	−21	11.82	552
	R	54	−3	−24	11.05	315

*MPFC, medial prefrontal cortex; PCC, posterior cingulate cortex; AG, angular; MTG, middle temporal gyrus*.

### FC Within DMN

**Figure 3:**
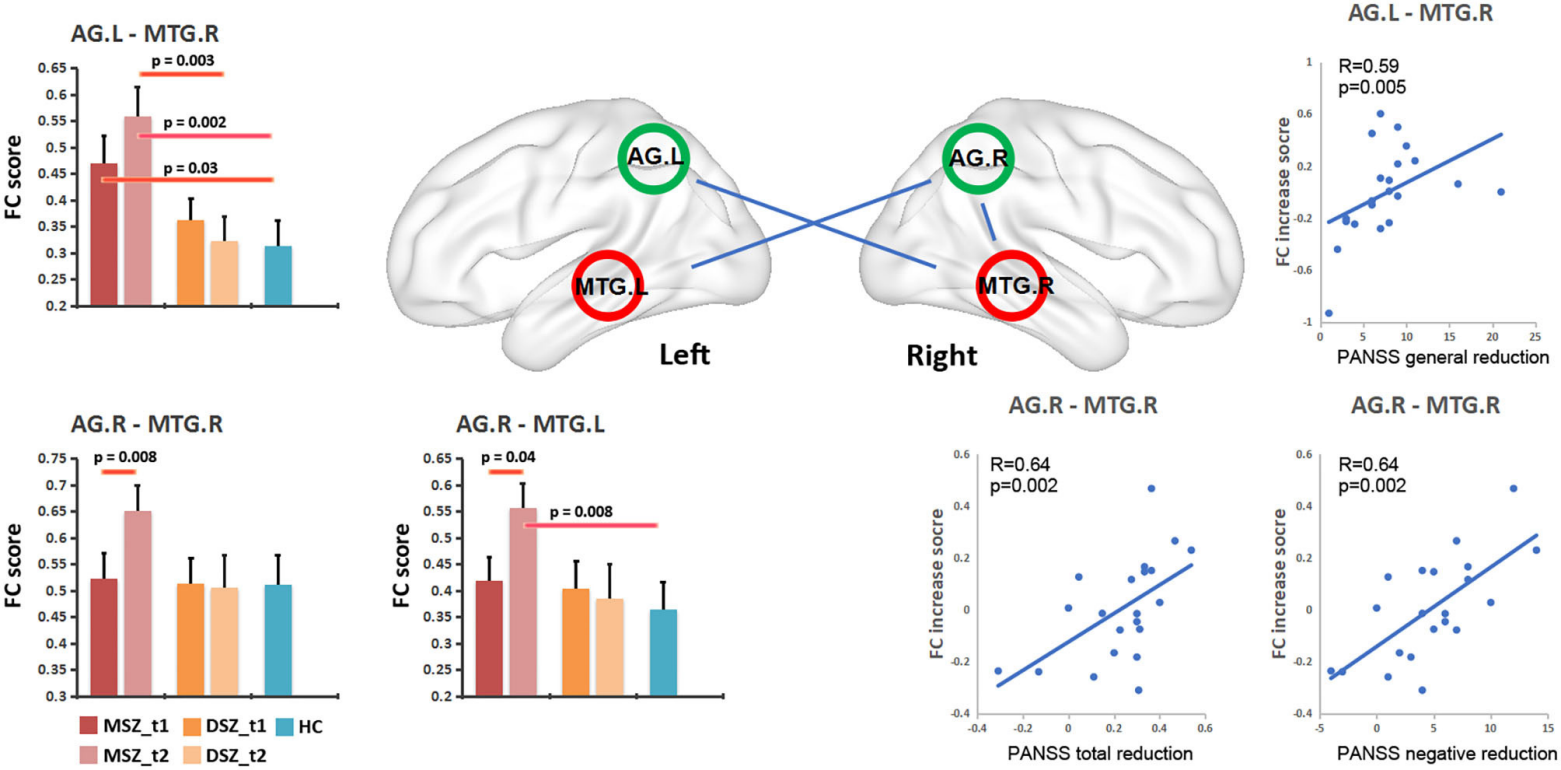
Changes of FC within the DMN in schizophrenia patients and relationship between FC change and PANSS reduction in the MSZ group. MSZ_*t*1, the patient group before ECT; MSZ_*t*2, the patient group after ECT; DSZ_*t*1, the patient group before only antipsychotic drug treatment; MSZ_*t*2, the patient group after only antipsychotic drug treatment; HC, healthy control group; NE, PANSS negative score; GE, PANSS general psychopathology score.

Three FCs of AG.L-MTG.R, AG.R-MTG.R, and AG.R-MTG.L showed the statistical significance (Figure 3). Significant group effect (*F* = 10.8, *p* = 0.002) was found for the FC of AG.L-MTG.R by the linear mix model after controlling for age, sex and chlorpromazine equivalent.

(1). *Group differences between MSZ and DSZ:* For the FC of AG.L-MTG.R, the MSZ group exhibited higher FC than the DSZ group at *t*2 (*T* = 3.16, *p* = 0.003), and not at *t*1, as well as the FC of AG.R-MTG.L (*T* = 2.3, *p* = 0.02). In the longitudinal analysis, the MSZ group showed increased FC after treatment in the FC of AG.L-MTG.R (*T* = 2.9, *p* = 0.008) and AG.R-MTG.L (*T* = 2.2, *p* = 0.04).
(2). *Group differences between MSZ/DSZ and HC:* For the FC of AG.L-MTG.R, the MSZ group showed higher FC than the HC group at *t*1 (*T* = 2.3, *p* = 0.03) and *t*2 (*T* = 3.4, *p* = 0.002). Besides, compared with the HC group, after treatment the MSZ group had greater FCs in AG.R-MTG.L (*T* = 2.3, *p* = 0.005) and AG.L-MTG.R (*T* = 2.1, *p* = 0.04). Additionally, no significant difference in FC between DSZ group and HC within DMN was found in *t*1 or *t*2.

### Relationships Between Connectivity Alterations and Clinical Features

Three FCs showed significant association with symptom remissions in the MSZ group after regressing out the age, gender and ECT number (Figure 3). After multiple comparisons correction by the family wise error (*P* < 0.05, FWE corrected), the FC change in AG.R-MTG.R was positively associated with a reduction of the PANSS negative score (*R* = 0.639, *p* = 0.002), and FC change in AG.R-MTG.R showed a positive association with the post treatment reduction ratio of PANSS total score (*R* = 0.64, *p* = 0.002). Moreover, the FC change in AG.L-MTG.R exhibited a significantly positive correlation with the reduction of PANSS general score (*R* = 0.59, *p* = 0.005).

## Discussion

This research project adopted a longitudinal experimental design to comprehensively evaluate the effects of ECT on resting state FC within the DMN in patients with SCZ treated conjointly with antipsychotics, as compared to a usual treatment group receiving antipsychotics. Our results demonstrated that, compared to the DSZ group, the MSZ group exhibited significantly increased FC between the bilateral AG and MTG after a course of ECT, thus indicating increased FC within the DMN. Furthermore, significantly positive correlations were noted between increased FC within the DMN and the reduction in PANSS scores in the MSZ group. These findings support the existence of a mechanism whereby ECT regulates the DMN in patients with SCZ, thus suggesting a potential relationship between ECT inducing hyperconnectivity within DMN and improvements in the symptomatology of SCZ.

Accumulating studies have reported the occurrence of abnormal FC within the DMN of SCZ patients (38, 39). However, these findings remain highly controversial due to heterogeneity of patients grouping and methods. Previous studies have found hyperconnectivity or hypoconnectivity of the DMN in SCZ patients, highlighting the need for separate consideration of different SCZ subtypes or stages of illness. For example, Fornito et al. suggested that increased FC is more prominent in early stages of SCZ, whereas decreased FC is characteristic of later stages of the disease (40). The initial FC increase may reflect an early compensatory activity increase in the central region, while the later decline could reflect decompensation or a later deterioration of function. Our present results showed a significantly increased FC in AG.L-MTG.R in the MSZ group at *t*1 compared to healthy controls, whereas there was no such increase in the DSZ group. Because the grouping of patients was non-random, this difference may reflect a slight difference in baseline clinical symptoms between the two groups, possibly due to poor drug response to drug-resistant SCZ in the MSZ group. However, the results are consistent with a specific effect of ECT in conjunction with medical treatment as usual.

Our results indicated hyperconnectivity within the DMN following ECT in the MSZ group, but not in the antipsychotics alone treatment group. The patients were all chronically medication-resistant SCZ patients, suggesting that there should be no pronounced changes in connectivity due to treatment as usual. Meanwhile, ECT did not reverse the original state of high connectivity, but provoked a compensatory increase. These results suggested that ECT can modulate the coordination of neuronal activity in spatially separated brain regions within the DMN, which may be the physiological mechanism underlying the regulation of brain function by ECT in these patients. The current results are also highly consistent with a previous study showing a functional shift of connectivity within the DMN after ECT treatment of treatment-resistant MDD patients. Specifically, the typically reduced DMN activation present in MDD patients was no longer present in the individuals who responded clinically to the ECT, but persisted in non-responders (41, 42). Thus, in distinct psychiatric disorders, clinical response to ECT may arise due to a common mechanism of increased connectivity within the DMN.

Previous evidence has shown that ECT can reduce the symptoms of SCZ by affecting local metabolism, dopamine signaling, and functional changes in brain regions (15, 43). Indeed, the present results of increased FC within the DMN following ECT are consistent with our hypothesis, and are in general agreement with results of our previous similar study using the same data (21). In that study, ECT combined with antipsychotic treatment appeared to increase global FC density within the precuneus (Pcu), ventral medial pre-frontal cortex (vMPFC), and dorsal medial pre-frontal cortex (dMPFC) regions, which are mainly located within the DMN. In our study, we also saw increased FC between the bilateral AG and MTG. This may reflect our decision to apply bitemporal stimulation delivery, which may be apt to alter connectivity specifically of the temporal lobe. As reported in previous studies, the placement of ECT electrodes can influence the efficacy of treatment, as well as effects on brain structure (44–46). Specifically, gray matter volume increases were observed in the bilateral hippocampus and left MTG after 6 weeks of ECT combined with antipsychotic treatment in patients with SCZ (47). Similar findings were found in a observer-independent coordinate-based meta-analysis of a large sample of MDD. An increase in the volume of the MTG was observed across different samples, sites, and processing pipelines after ECT (48). Another MDD study showed that the main and side effects of ECT would lead to increased volumes of the hippocampus and amygdala in the affect processing network, which seems to be a plausible antidepressant mechanism that could explain the efficacy of ECT (49). This suggests that studies of structural changes in the brain may provide more meaningful explanations of the therapeutic neuromechanism of ECT. Additionally, previous studies have shown that various parts of the left anterior temporal lobe (ATL), posterior middle temporal gyrus (pMTG), and AG are candidates for higher-order convergence zones or network hubs of semantic processing (50, 51). A previous study of inhibitory TMS in healthy controls showed that stimulation to the AG interfered with the automatic retrieval of specific concepts from the semantic store, while stimulation of pMTG impaired semantic cognition. Therefore, we speculate that the hyperconnectivity between MTG and AG seen in this study may reflect compensatory changes related to restoration of semantic processing function in SCZ patients to the normal state, in concert with alleviation of certain clinical symptoms. As such, our findings provide important new insights into the therapeutic effects of ECT on SCZ.

In addition, the present study demonstrated a significant positive correlation between the reductions in PANSS symptom subscales (negative, general and total) and increased FC within the DMN in the MSZ group, indicating that the improvement in SCZ negative symptoms may be associated with hyperconnectivity within the DMN. The correlations between increased FC with improved clinical symptoms of SCZ thus constitute evidence for a mechanism whereby ECT can influence the symptomatology of SCZ.

The present study has several limitations, notably in relation to the relatively small sample size. Indeed, this study is a preliminary study on the neural mechanism of ECT, and we are confident that more convincing results will be obtained in the future with a larger sample size. In addition, there were some differences in medication regimens between the groups, which may have caused some potential effects on the results. Second, our study only analyzed functional changes in DMN after ECT, and we will incorporate brain structural data into the study in the future. Thirdly, the patients in the MSZ group were taking antipsychotic medications while receiving ECT, thereby introducing a confounding factor in the simple interpretation of our results. Additionally, since the patients were non-randomly allocated to the ECT treatment, there arises some possible discrepancy in results between the MSZ and DSZ groups at *t*1. However, our study indicates that ECT combined with antipsychotic therapy might contribute to the improvement of abnormal symptomatology via inducing a plastic restoration of DMN connectivity in SCZ patients, perhaps in relation to neurotrophic effects of the treatment in interaction with antipsychotic medication. Finally, the seed-based approach was used in our study to obtain the corresponding DMN. Although the seed-based approach is simple and effective, it is hypothesis-driven and requires prior knowledge of the seed to demonstrate that representativeness of ROI is not always reliable. However, using brain atlas or data-driven independent components analysis to extract the time series in DMN may only yield the mean time series of DMN, which is often used when calculating FC between the DMN and other networks. In our study, we aimed to calculate FC between nodes in DMN by extracting time series of these key nodes, so as to construct functional connection network within DMN. In future work, we can also try to divide DMN into multiple components by ICA method to analyze the connectivity between components.

In conclusion, our study provides further evidence for ECT-induced increases in FC within the DMN in patients with SCZ (Figure 3). Furthermore, the response of hyperconnectivity within the DMN correlated with the individual therapeutic response in patients with SCZ. These findings confirmed a potential relationship between ECT inducing hyperconnectivity within DMN and improvements in symptomatology of SCZ, suggesting that ECT controls mental symptoms by regulating the temporoparietal connectivity within DMN.

## Data Availability Statement

The original contributions presented in the study are included in the article/supplementary material, further inquiries can be directed to the corresponding authors.

## Ethics Statement

The studies involving human participants were reviewed and approved by the Ethics Committee of SMHC approved the study protocol; all subjects provided written, informed consent prior to participation in the study. The patients/participants provided their written informed consent to participate in this study. Written informed consent was obtained from the individual(s) for the publication of any potentially identifiable images or data included in this article.

## Author Contributions

QH and HH conceived of the study, participated in the clinical treatment, and helped to draft the manuscript. YJ, XJ, and JZ collected the data and carried out the MRI assessment and imaging data acquisition. YT and TZ contributed toward data analysis. JS, DY, and CLu helped to revise the manuscript. CLi and JW are the guarantors of integrity of the entire study. All authors read and approved the final manuscript.

## Funding

This work was supported by National Natural Science Foundation of China grants (81971251, 81671329, 81871050, 82171497, 82101582, 82001406); Clinical Research Center at Shanghai Mental Health Center grants (CRC2018ZD01, CRC2019ZD02, CRC2018ZD04, CRC2018YB01); Clinical Research Center at Shanghai Jiaotong University School of Medicine (DLY201817); Shanghai Science and Technology Committee Foundations (19411950800, 16ZR1430500, 19411969100, 19410710800, 21ZR1481500, 20ZR1448600, 21S31903100, 19ZR1445100); Shanghai Clinical Research Center for Mental Health (19MC1911100); Project of the Key Discipline Construction, Shanghai 3-Year Public Health Action Plan (GWV-10.1-XK18); Shanghai Municipal Science and Technology Major Project (No.2018SHZDZX01, 2018SHZDZX05) and ZJLab; Foundation of Shanghai Mental Health Center(2020-FX-02).

## Conflict of Interest

The authors declare that the research was conducted in the absence of any commercial or financial relationships that could be construed as a potential conflict of interest.

## Publisher's Note

All claims expressed in this article are solely those of the authors and do not necessarily represent those of their affiliated organizations, or those of the publisher, the editors and the reviewers. Any product that may be evaluated in this article, or claim that may be made by its manufacturer, is not guaranteed or endorsed by the publisher.
